# Intraprostatic locations of tumor foci of higher grade missed by diagnostic prostate biopsy among potential candidates for active surveillance

**DOI:** 10.1038/srep36781

**Published:** 2016-11-09

**Authors:** Kwangmo Kim, Jung Keun Lee, Gheeyoung Choe, Sung Kyu Hong

**Affiliations:** 1 Department of Urology, Seoul National University Bundang Hospital, Seongnam, Korea; 2 Department of Pathology, Seoul National University Bundang Hospital, Seongnam, Korea

## Abstract

To establish optimal biopsy scheme for selection of candidates for active surveillance (AS) among prostate cancer (PCa) patients, information on topographical distribution of tumor foci of higher grade missed by contemporary biopsy amongst potential candidates of AS would certainly be useful. Thus we analyzed topographic distribution of tumor foci by examining prostatectomy specimens in 444 patients who underwent radical prostatectomy for low risk PCa. Anterior and posterior prostate areas were demarcated by a horizontal line drawn at midpoint of prostatic urethra. Among 444 subjects, patients with upgrading showed relatively higher prevalence of index tumor foci in anterior prostate than those without upgrading, though not reaching statistical significance (p = 0.252). Meanwhile, among 135 (30.4%) patients with very low risk PCa, patients with upgrading showed significantly higher prevalence of index tumor foci in anterior prostate than those without upgrading (52.2% vs 33.8%; p = 0.031). In conclusions, tumor foci of higher grade missed by diagnostic biopsy were mostly located in anterior prostate among very low risk PCa patients. Such finding would be concrete evidence to support the notion that more efforts are needed to increase accuracy in detecting tumor foci in anterior prostate among potential candidates for AS.

Currently, active surveillance (AS) is widely accepted as a treatment option for low risk prostate cancer (PCa)^1^. Meanwhile, a proportion of men clinically diagnosed with low risk PCa actually harbor higher-grade disease necessitating radical treatment. Published data have shown that Gleason score upgrading occurs in 30% to 50% of patients with low risk disease undergoing radical prostatectomy (RP)^2^,^3^. Efforts have been made to develop useful tools for the prediction of upgrading among men deemed suitable for AS. Different institutions use different tools and criteria in the selections of candidates for AS^4^,^5^. Accurate identification of patients with indolent disease remains a significant challenge in the implementation of AS program.

To establish an optimal prostate biopsy scheme for selection of appropriate candidates for AS, information on the topographical distribution of tumor foci of higher grade missed by contemporary biopsy scheme amongst potential candidates of AS would certainly be important. Although various nomograms and tools, including MRI, have been reported to enhance the prediction of upgrading, their diagnostic accuracy varies with none being perfect in differentiating indolent from more aggressive tumors^6^,^7^. Also published data on the actual intraprostatic distribution of tumor foci of higher-grade (≥Gleason grade 4) missed among patients initially diagnosed with low risk PCa are scarce. Thus, we analyzed the topographical distribution of tumor foci in patients with low risk PCa who underwent RP.

## Patients and Methods

### Subjects

With the approval of our institutional review board, we retrospectively reviewed the medical records of 1,822 patients who underwent RP at a single institution from July 2006 to December 2013. After exclusion of 279 patients (neoadjuvant hormonal therapy [n = 40], insufficient medical record (referred patients who had biopsy at other hospital) [n = 239]), we stratified the 1543 subjects into three risk groups according to D’Amico risk criteria. Overall a total of 444 patients who were revealed to have the low risk PCa (clinical stage T1c to T2a, biopsy Gleason score six or less, serum prostate specific antigen (PSA) <10 ng/ml) were finally included in our analysis^8^. Among the 444, 135 (30.4%) men had very low risk PCa (clinical stage T1c, biopsy Gleason score six or less, prostate specific antigen density (PSAD) <0.15, 2 or fewer positive biopsy cores, and 50% or less cancer involvement per core)^1^. The preoperative and postoperative information such as biopsy data, PSA level, clinical stage and pathologic outcomes were assessed by the review of medical records. As the biopsy Gleason score of the entire subjects were ≤6, the pathologic upgrading was defined as any pathologic Gleason score ≥7. The pathological stage was evaluated according to the 2010 WHO TNM staging system.

The study was performed in accordance with the standards of the Declaration of Helsinki, with a waiver of informed consent because of its retrospective fashion. The study protocol was reviewed and approved by the institutional Ethics Committee of the Seoul National University Bundang Hospital, Korea(IRB number: B-1606-349-116). This research with all experimental protocol was carried out in accordance with the approved guidelines and the guidelines verified and approved by the institutional Ethics Committee of the Seoul National University Bundang Hospital.

### Pathologic evaluation of the postoperative specimens

The pathologic specimens were fixated in 10% buffered formalin for 24 hours and laminated in 3 millimeters slices along the coronal plane from apex to base. Each apex and base slice was laminated vertically for evaluation of the margin involvement by the tumor. In each slice, all tumor focus was configurated. Longitudinally from apex to base, the lower one-third slices nearby apex were defined as low body, mid one-third slices as mid body and the upper one-third slices as high body. Also, when tumor was located anterior to the horizontal line drawn at the midpoint of prostatic urethra, its location was designated as anterior prostate, and when located posterior to the horizontal line, posterior prostate. Since tumor foci can extend across more than one sector, the locations of index tumor foci were designated as the sectors where largest proportions of foci were observed to be located. For a given index tumor focus, its location was designated as one of aforementioned longitudinal sectors and also as one of transverse sectors (anterior or posterior). Therefore the tumor location was categorized as apex, low body, mid body, high body, or base in longitudinal plane and anterior or posterior in transverse plane. Single experienced pathologist reviewed the pathological specimens and recorded the number, volume, Gleason pattern and the location of each tumor foci. The index tumor was defined as tumor focus with the highest Gleason score. If there were multiple tumor focus with same highest Gleason score, the largest tumor focus was determined as the index tumor.

### Statistical analyses

The chi-square tests and student t-tests were utilized to compare the differences between the subgroups. The logistic regression tests were used for uni- and multi-variate analyses. All statistical analyses were performed by SPSS version 21.0 (IBM, Chicago, IL, USA). All p-values were two-sided and values <0.05 were considered statistically significant.

## Results

### Patient characteristics

The clinical and pathologic characteristics of 444 low risk prostate cancer patients were summarized in Table 1. There were 307 patients (69.1%) who presented the upgrading of Gleason score after RP from biopsy Gleason 6 to pathologic Gleason score 7 or higher. When we compared the preoperative characteristics between the two subgroups divided according to the presence of Gleason score upgrading after RP, there were no significant difference in age, preoperative PSA, and clinical stage (all *P* values > 0.05). But patients with upgrading showed significantly higher PSA density (*P* < 0.001), longer tumor length in biopsy core (*P* < 0.001), and higher number of positive biopsy cores (*P* < 0.001) than the patients without upgrading. In addition, the patients with Gleason score upgrading also showed worse pathological outcomes than the patients without upgrading. The pathological stage (*P* < 0.001), the rate of surgical margin involvement (*P* < 0.001), and the total tumor volume (*P* < 0.001) were significantly higher. Among the 135 men with very low risk PCa, similar trends were observed in comparing those with and without upgrading. Very low risk patients with upgrading had longer tumor length in biopsy core (*P* = 0.004) and larger tumor volume in RP specimen (*P* < 0.001) than those without upgrading.

### Topographic analyses

**Figure 1:**
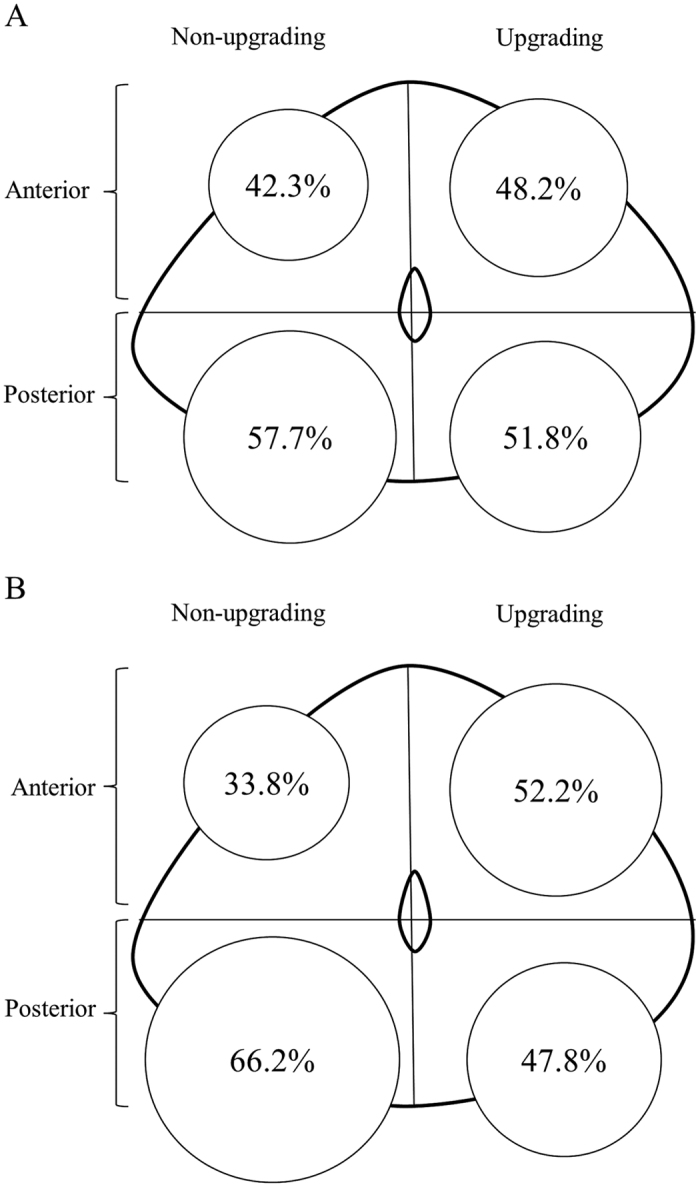
Respective prevalences of index tumor foci in anterior and posterior prostate among patients with and without upgrading after radical prostatectomy. Low risk group (**A**), very low risk group (**B**).

When we compared the locations of index tumor foci (tumor foci with highest Gleason score and/or largest tumor volume) between patients with and without Gleason score upgrading among our 444 subjects, the patients with upgrading showed higher rate of index tumor detections in high body (*P* = 0.002) and base (*P* = 0.023) among longitudinal sectors of prostate (Table 2A). Also patients with Gleason score upgrading showed relatively higher rate of index tumor detections in anterior prostate than those without upgrading, not reaching statistical significance (48.2% vs 42.3%; *P* = 0.252) (Fig. 1A). When we analyzed the percentage of index tumor with larger volume (≥0.5 cm^3^), the patients with Gleason score upgrading revealed to have higher proportion of such larger index tumor (67.2% vs 27.0%; *P* < 0.001). Among only the 135 very low risk group, similar trends were observed. Very low risk patients with upgrading had higher rates of index tumor detections in high body (*P* = 0.039) among longitudinal sectors (Table 2B). Meanwhile, most notably, very low risk patients with upgrading were observed to have significantly higher rate of index tumor detections in anterior prostate than those without upgrading (52.2% vs 33.8%; *P* = 0.031) (Fig. 1B).

### Predictors of upgrading

We performed multivariate analyses to identify potential predictors of Gleason score upgrading among patients with low risk PCa (Table 3). Our multivariate analyses revealed that patient age (*P* = 0.008), PSA density (*P* = 0.004), number of positive cores (*P* = 0.027), and tumor length in biopsy core (*P* = 0.003) were significantly associated Gleason score upgrading. When the same analyses were performed among the very low risk group, only tumor length in biopsy core was observed be to a significant preoperative predictor of Gleason score upgrading (*P* = 0.021).

## Discussion

By performing topographical histopathologic analyses of RP specimens in this study, we observed that patients with upgrading after RP showed significantly higher rate of index tumor foci localization in anterior prostatic sector compared with those without upgrading among the patients with very low risk PCa who are widely considered appropriate candidates for AS. Such trend was also found among the low risk group as a whole, though not reaching statistical significance. Despite the fact that PCa tumor foci are prone to be located in peripheral zone, we observed that about half of index tumor foci in low risk PCa patients who had upgrading after RP were actually located in anterior prostate. Such findings would be concrete evidence to support the notion that more efforts are needed to increase the accuracy in detecting tumor foci in anterior prostatic area by TRUS-guided biopsy, especially among men who are clinically deemed appropriate for undergoing AS. In this study, we also confirmed that patients with upgrading generally had worse pathologic features.

Currently, a paucity of data exists on the actual intra-prostatic locations of tumor foci of higher grade missed by conventional TRUS-guided biopsy in low risk PCa patients. Using a data-acquisition model storing graphic and textual clinical information, Eminaga *et al.* reviewed 168 consecutive RP specimens to analyze the distribution of PCa foci^9^. They found that tumor foci with Gleason score 6 were mostly concentrated in the posterior part of peripheral zone of prostate, whereas PCa foci with Gleason score >6 extended towards the base and anterior parts of prostate. Although their subjects were not limited to low risk group upgraded after RP, such findings would be supportive of our results as index tumor foci were shown to be located in anterior prostate more frequently among patients with upgrading (Gleason score >6) than those without upgrading (Gleason score 6) in our study. In another study, the same group also reported that preoperative serum PSA levels varied according to the topographical distribution of PCa in RP specimens as they observed that PCa with PSA level 10.1–20 ng/ml was found more frequently in anterior part and base of prostate than PCa with PSA level <10 ng/ml^10^. However, contradictory findings have been reported previously by others^11^. In the current study, we could not confirm higher probability of anterior cancer showing higher PSA level (data not shown). It is likely that factors other than location of tumor foci, such as prostate volume (transitional and peripheral zone) and volume of tumor foci of different grade, may well have contributed to overall PSA level. Although not on tumor grade, Davis *et al.* reviewed RP specimens of 66 patients who met AS selection criteria and concluded that tumor foci of transition zone origin contributed to underestimated tumor volume in a significant number of cases^12^. Also Sundi *et al.* evaluated RP specimens in 87 black and 89 white men with very low risk PCa and reported that black men with such disease have a significantly higher prevalence of anterior cancer foci that are of higher grade and larger volume than white counterparts^13^. In men with upgrading after RP, they observed that dominant nodule was more frequently anterior in black than in white men (59% vs 0%, respectively). Considering such results along with our findings, the possibility of racial difference can be suggested regarding the topographical distribution of PCa within prostate. As 52.2% of our very low risk group with upgrading had dominant nodule located in anterior prostate, such rate may be considered similar to the aforementioned rate of anterior tumor nodule in black patients reported by Sundi *et al.*^13^. On the other hand, the technical differences in topographic analyses should be considered as some of anterior tumor foci assessed in our study included tumor foci also extending into posterior prostate. As several groups reported on the observed differences between PCa in black and white men, comparative investigations encompassing Asian PCa patients are also warranted.

As tumors in anterior prostate cannot be palpated and poorly localized via TRUS, it is plausible to assume that tumor foci in anterior prostate would frequently be undetected by initial TRUS-guided biopsy. Previously, others have also reported upon relative difficulty of detecting anterior tumor. Bott *et al.* found that anterior tumors required more biopsy sessions to detect than posterior tumors^14^. Unlike most relevant studies on anterior tumors of prostate, it should be reminded that our study focused on the actual intraprostatic locations of tumor foci of higher grade missed by initial biopsy among patients diagnosed with Gleason 6 PCa from initial biopsy. Our findings indicate that a change in strategy is needed for a more accurate depiction of disease in potential candidates of AS. Currently MRI-targeted biopsy has been reported to be useful in detection of evasive anterior tumors^15^. A review of current literature supporting utility of multi-parametric MRI (mpMRI) showed the sensitivity of mpMRI for PCa detection to be 80–90% and the specificity for suspicious lesion to be between 50% and 90%^16^. Despite a growing body of literature, debate over capability of mpMRI in reliably detecting significant cancer still remains^17^. Others have advocated saturation biopsies to enhance cancer detection rate. Motamedinia *et al.* reported that near saturation biopsies with a mean of 17 cores before starting AS detected previously missed high grade tumor foci in more than 70% of low risk cases^18^. A computer simulation study has demonstrated that template mapping biopsies (TMB) in which a median of 48 cores were obtained via transperineal approach outperformed standard 12-core TRUS-guided biopsy for detection of clinically significant PCa^19^. The same simulation study showed that adding more anterior cores to TRUS-guided biopsy would only provide marginal improvement over standard TRUS-guided biopsy, also being inferior to performance of TMB. Also Barzell *et al.* observed that repeat TRUS-guided biopsy failed to detect up to 80% of clinically significant tumors detected by TMB^20^. They suggested that TMB would enhance detection of anterior tumors compared with TRUS-guided biopsy. Meanwhile, others have raised questions regarding TMB, citing higher cost and procedural issues^21^. Although MRI-targeted and transperineal saturation biopsies are not without downside, they may indeed be appropriate for potential candidates of AS.

Our study may be limited by the retrospective nature. However, the risk of selection bias can be considered as being lower than similar western series from contemporary period since AS for low risk PCa was not performed widely in Korea during the study period. Admittedly, low risk PCa patients who opted for non-surgical treatment, such as radiation therapy, could not be included in our study.

## Conclusions

In our study, we observed that tumor foci of higher grade missed by diagnostic biopsy were mostly located in anterior prostate among the patients with very low risk PCa who are widely considered appropriate candidates for AS. Patients with upgrading after RP showed significantly higher rate of index tumor foci localization in anterior prostate compared with those without upgrading. Similar trend was also noted among the low risk group as a whole with about half of index tumor foci in low risk PCa patients with upgrading after RP located in anterior prostate. Such findings would be concrete evidence to support the notion that more efforts are needed to increase the accuracy in detecting tumor foci in anterior prostatic area by TRUS-guided biopsy, especially among men who are clinically deemed appropriate for undergoing AS. Our findings should be considered in efforts to optimize prostate biopsy scheme for the selection of appropriate candidates for AS.

## Additional Information

**How to cite this article**: Kim, K. *et al.* Intraprostatic locations of tumor foci of higher grade missed by diagnostic prostate biopsy among potential candidates for active surveillance. *Sci. Rep.* **6**, 36781; doi: 10.1038/srep36781 (2016).

**Publisher’s note:** Springer Nature remains neutral with regard to jurisdictional claims in published maps and institutional affiliations.

## Figures and Tables

**Table 1:** Comparison of patients with and without Gleason score upgrading on pathologic examination of radical prostatectomy specimen.

	Gleason score non-upgrading	Gleason score upgrading	*P* value
Number of patients	137	307	
Mean age (years)	64.7 ± 6.8	65.7 ± 6.6	0.178
Mean BMI (kg/m^2^)	24.2 ± 2.4	24.2 ± 27	0.924
Mean PSA (ng/ml)	5.4 ± 2.1	5.7 ± 1.9	0.304
Mean PSAD (ng/ml/cc)	0.13 ± 0.06	0.18 ± 0.09	<0.001
Mean prostate volume (cc)	44.2 ± 16.8	36.2 ± 15.3	<0.001
Clinical stage (%)			
T1	113 (82.5)	243 (79.2)	0.240
T2	24 (17.5)	64 (20.8)	
Mean number of positive core	1.8	2.8	<0.001
Mean tumor length in biopsy core (cm)	0.22 ± 0.19	0.39 ± 0.29	<0.001
Pathologic stage (%)			
T2	133 (97.1)	271 (88.3)	<0.001
T3	4 (2.9)	36 (11.7)	
Positive surgical margin (%)	5 (3.6%)	55 (17.9%)	<0.001
Mean total tumor volume (cc)	0.6 ± 0.9	2.2 ± 2.6	<0.001
Mean index tumor volume (cc)	0.5 ± 0.8	1.6 ± 1.9	<0.001
Mean number of tumor foci	2.7 ± 1.6	3.6 ± 1.9	<0.001

BMI = body mass index, PSA = prostate-specific antigen, PSAD = prostate-specific antigen density.

**Table 2:** Intra-prostatic locations of index tumor foci among low risk (A) and very low risk patients (B).

	Gleason score non-upgrading	Gleason score upgrading	*P* value
**(A) Low risk patients (N = 444)**			
Anterior	58 (42.3)	148 (48.2)	0.252
Posterior	79 (57.7)	159 (51.8)	0.252
Apex	11 (8.0)	2 (0.7)	<0.001
Low body	48 (35.0)	64 (20.8)	0.001
Mid body	55 (40.1)	131 (43.0)	0.574
High body	22 (16.1)	93 (30.3)	0.002
Base	1 (0.7)	16 (5.2)	0.023
**(B) Very low risk patients (N = 135)**			
Anterior	23 (33.8)	35 (52.2)	0.031
Posterior	45 (66.2)	32 (47.8)	0.031
Apex	8 (11.8)	1 (1.5)	0.017
Low body	30 (44.1)	22 (32.8)	0.178
Mid body	25 (36.8)	29 (43.3)	0.440
High body	5 (7.4)	13 (19.4)	0.039
Base	0 (0.0)	2 (3.0)	0.151

**Table 3:** Multivariate logistic regression analysis of preoperative factors associated with Gleason score upgrading in low risk (A) and very low risk patients (B).

	OR (95% CI)	*P* value
**(A) Low risk patients**		
Age	1.047 (1.012–1.083)	0.008
PSAD × 100	1.059 (1.018–1.103)	0.004
Prostate volume	0.988 (0.973–1.003)	0.122
Number of positive core	1.216 (1.022–1.446)	0.027
Mean tumor length in biopsy core	6.537 (1.928–22.158)	0.003
**(B) Very low risk patients**		
Age	1.035 (0.978–1.095)	0.234
PSAD × 100	0.975 (0.844–1.127)	0.733
Prostate volume	1.001 (0.978–1.025)	0.907
Number of positive core	1.573 (0.704–3.517)	0.270
Mean tumor length in biopsy core	14.568 (1.498–141.662)	0.021

CI = confidence interval, OR = odds ratio, PSAD = prostate-specific antigen density.
